# The ACE2 decoy receptor can overcome immune escape by rapid mutating SARS-CoV-2 variants and reduce cytokine induction and clot formation

**DOI:** 10.1186/s12929-025-01156-4

**Published:** 2025-06-26

**Authors:** Ming-Shiu Lin, Tai-Ling Chao, Yu-Chi Chou, Yao Yi, Ci-ling Chen, Kuo-Yen Huang, Sui-Yuan Chang, Pan-Chyr Yang

**Affiliations:** 1 Department of Internal Medicine, National Taiwan University College of Medicine, Taipei, Taiwan; 2 Institute of Biomedical Sciences, Academia Sinica, Taipei, Taiwan; 3 Department of Clinical Laboratory Sciences and Medical Biotechnology, National Taiwan University College of Medicine, Taipei, Taiwan; 4 Biomedical Translation Research Center (BioTReC), Academia Sinica, Taipei, Taiwan; 5 Graduate School of Advanced Technology (Program for Precision Health and Intelligent Medicine), National Taiwan University, Taipei, Taiwan; 6 National Taiwan University YongLin Institute of Health Scholar, Taipei, Taiwan; 7 Department of Laboratory Medicine, National Taiwan University Hospital, Taipei, Taiwan; 8 Genomics Research Center, Academia Sinica, Taipei, Taiwan

**Keywords:** ACE2-Fc, SARS-CoV-2, Spike, Immune escape

## Abstract

**Background:**

The COVID-19 pandemic continues to affect the world in 2025. The rapid mutation of SARS-CoV-2 results in breakthrough infections and diminishes the efficacy of vaccines and anti-viral drugs. The severity of the disease varies across different variants, and the underlying mechanisms driving these differences remain unclear. This study explores the relationship between different Spike variants and cytotoxicity, aiming to determine whether the humanized decoy receptor ACE2-Fc can neutralize spikes from diverse variants, offering a solution to overcome rapid mutating SARS-CoV-2 induced immune escape.

**Methods:**

We co-cultured 293 T-ACE2 cells with 293 T cells transfected with various Spike protein variants or used H1650-ACE2 cells transfected with these Spike variants. This allowed us to observe the effects of different Spike mutations, specifically focusing on cell fusion, cytotoxicity, and cytokine release from human peripheral blood mononuclear cells. Flow cytometry is employed to determine if ACE2-Fc can recognize different Spike variants. We also assess the ability of ACE2-Fc to inhibit infection, cell fusion, cytotoxicity, and cytokine release through pseudovirus infections or Spike protein transfections. Additionally, we use actual viruses from SARS-CoV-2 patients to validate the impacts of Spike mutations and the effectiveness of ACE2-Fc. Furthermore, human plasma is utilized to evaluate ACE2-Fc’s capability to inhibit Spike-induced clot formation.

**Results:**

We found that different Spike variants, particularly those with enhancements at the S2' site, increased cell–cell fusion capability, which correlated positively with cytotoxicity and cytokine IL-6 and TNF-α released from PBMCs. ACE2-Fc recognized spikes from wide-range of variants, including wild type, Alpha, Delta, Delta plus, Lambda, BA.2, BA.2.75, BA.5, BF.7, BQ.1, XBB.1, JN.1, KP.2, and KP.3, and effectively prevented these spike-expressing pseudo-viruses from entering host cells. Crucially, ACE2-Fc can prevent spike-induced cell fusion, thereby reducing subsequent cytotoxicity and the release of IL-6 and TNF-α from PBMCs. ACE2-Fc also effectively reduces plasma clot formation induced by trimeric spike proteins.

**Conclusions:**

These findings demonstrated that ACE2-Fc could effectively combat the infection of rapidly mutating SARS-CoV-2, providing a potential solution to overcome immune evasion.

**Supplementary Information:**

The online version contains supplementary material available at 10.1186/s12929-025-01156-4.

## Background

SARS-CoV-2, the virus responsible for COVID-19, known for their single-stranded RNA structure, is classified within the coronavirus family. This virus was first identified in late 2019, emerging as a new member in a lineage of zoonotic coronaviruses, which includes the SARS-CoV outbreak in 2002 and the MERS-CoV outbreak in 2012. Unlike its predecessors, SARS-CoV-2 demonstrated a significantly higher transmission rate, rapidly spreading across the globe, resulting in widespread infection and substantial loss of life [1–3]. The ongoing pandemic continues to pose severe challenges to global health systems, as the virus evolves into new variants that complicate efforts to control its spread and mitigate its impact [4, 5].

The disease progression of COVID-19 involves a complex interaction between the SARS-CoV-2 virus and the human immune system. The severity of the disease, especially in severe cases, is primarily due to an overactive immune response rather than the virus itself [6]. This response includes a cytokine storm, characterized by high levels of cytokines like IL-1β, IL-6, GM-CSF, IFN-γ, and TNF-α, which cause widespread inflammation and affect multiple organs [7, 8]. A critical element in COVID-19 severity is IL-6, a cytokine crucial in managing inflammatory responses. During an infection, immune cells such as macrophages, neutrophils, dendritic cells, and lymphocytes produce excessive IL-6. This overproduction contributes to the cytokine storm and is linked to more severe disease and poorer outcomes [9–11]. However, the exact mechanisms by which the virus triggers this excessive cytokine release are still under investigation.

The Spike (S) protein of SARS-CoV-2 is vital for the its ability to enter host cells, making it a key target for COVID-19 therapeutics such as vaccines and antibody treatments. The Spike protein uses the ACE2 receptor to gain entry to the host cells, and its rapid mutation, especially in the receptor binding domain (RBD), presents significant challenges [3, 12]. These mutations can increase the Spike protein’s ability to bind to ACE2, enhance the infectivity of various viral strains and altering the severity of the disease in patients. Furthermore, these mutations contribute to immune escape by altering the epitopes targeted by neutralizing antibodies. This change can reduce the effectiveness of these antibodies, which are essential for immune defense [13–15]. Consequently, the mutating Spike protein complicates ongoing efforts to manage the pandemic by decreasing the efficacy of existing vaccines and therapeutic antibodies.

We previously demonstrated that ACE2-Fc could block the original strain of SARS-CoV-2 and prevent cell fusion [16]. Subsequent research confirmed that using natural receptor as decoy, ACE2-Fc could also neutralize a wide range of mutant strains, including Alpha, Beta, Delta, Gamma, and Omicron B.1 [17–19], suggesting its potential as a universal therapeutic option. Various research groups have shown that different forms of ACE2 decoys are effective against SARS-CoV-2 in vivo. These decoys can reduce viral infection, prevent lung injury and edema, and improve survival in humanized mice, hamsters, and non-human primates [20–23]. Among them, ACE2-IgM has demonstrated good safety and promising antiviral activity in a Phase 1 clinical trial involving individuals infected with SARS-CoV-2 [24, 25]. These findings highlight the strong potential of ACE2 decoys as a therapeutic strategy against SARS-CoV-2. In this study we further expanded to show that different mutant strains of the Spike protein vary in their ability to induce cell fusion, cytotoxicity, and cytokine production, which may affect disease severity. Notably, ACE2-Fc not only prevented infections by different mutant variants but also reduced the associated cytotoxicity, inflammatory responses and clot formation. This evidence supports the potential of ACE2-Fc as a wide-range therapeutic agent against the ongoing evolution of SARS-CoV-2.

## Methods

### Generation of the ACE2-Fc fusion protein

We engineered an ACE2-Fc fusion protein by amplifying a key ACE2 segment (18‐615 A.A.) and integrating it with the Fc region of human IgG1 into pcDNA 3.1(–) vectors. Utilizing the ExpiCHO expression system, we produced and harvested the fusion protein from CHO cell culture medium. The protein was then purified using Protein G Sepharose and its quality was validated by measuring concentration with NanoDrop at 280 nm and assessing purity through polyacrylamide gel electrophoresis.

### Cell lines

Our research utilized various cell lines, including HEK293T, H1650, H1299, and Vero E6, all sourced from the American Type Culture Collection (ATCC), and human peripheral blood mononuclear cells (PBMCs) obtained from Lonza Bioscience. Standard culture conditions involved the use of RPMI 1640 medium or DMEM, enriched with 10% heat-inactivated fetal bovine serum and 1% penicillin–streptomycin. The cells were incubated in a controlled environment with 5% CO2 and 95% air. Additionally, ExpiCHO cells, acquired from Life Technologies, were grown in specialized ExpiCHO expression medium and maintained under constant agitation at 120 rpm and a temperature of 37 °C.

### Immunoblot

Cells were harvested and lysed using an IP lysis buffer composed of 20 mM Tris (pH 7.5), 100 mM sodium chloride, 1% IGEPAL CA-630, 100 µM Na3VO4, 50 mM NaF, and 30 mM sodium pyrophosphate, supplemented with Roche protease inhibitors. The protein concentrations were quantified using the BCA assay kit from Thermo Fisher Scientific. Samples containing equal amounts of protein were denatured and subjected to SDS-PAGE, followed by transfer to PVDF membranes from Millipore. These membranes were blocked with a 5% nonfat milk solution in PBS with 0.1% Tween-20 from Sigma Aldrich for 1 h. Subsequently, membranes were incubated with primary antibodies against the Spike protein (GeneTex, 1:1,000) and actin (Sigma, 1:10,000) overnight at 4 °C. Afterward, they were exposed to a horseradish peroxidase-conjugated secondary antibody for 1 h at room temperature. Signals were detected using enhanced chemiluminescence and captured with a ChemiDoc MP Imaging System by Bio-Rad.

### Lactate dehydrogenase (LDH) cytotoxicity assay

We assessed cell cytotoxicity using the LDH Cytotoxicity Detection Kit by Roche, following the provided guidelines. H1650-ACE2 cells were either transfected with a spike plasmid or infected with SARS-CoV-2 for 48 h. Post-treatment, we collected the supernatants to measure LDH activity, indicative of cell membrane integrity and thus, cytotoxicity. We used the medium from untreated cells as a low control (baseline LDH release) and from cells treated with a 2% Triton X-100 solution as a high control (maximum LDH release). For the assay, we mixed 100 µL of each supernatant with 100 µL of the reaction mixture and incubated this at room temperature for 1–2 h. We then measured the absorbance at 490 nm using a VersaMax microplate reader (Molecular Devices). The percentage of cytotoxicity was calculated using the formula:

$$\left( {{\text{Supernatant value}}\, - \,{\text{Low control}}} \right)/\left( {{\text{High control}}\, - \,{\text{Low control}}} \right)\, \times \,{1}00$$

### Syncytia formation

Following Huang et al. (2020), we examined syncytia formation using 293 T cells transfected with 5 µg of pCR3.1-Spike variants and 0.5 µg of pLKO AS2-GFP (Lipofectamine 3000, Thermo Fisher). After 2 days, these 293 T–S effector cells were treated with polyclonal human IgG (Jackson ImmunoResearch Laboratories) or ACE2-Fc for 1 h, then co-cultured with 293 T-ACE2 target cells for 24 h at 37 °C. Fusion events were visualized using a Zeiss Axiovert 200 M microscope, with green fluorescence quantified by MetaMorph software.

Additionally, H1650-ACE2 cells were transfected with 2 µg Spike plasmids for 6 h, followed by IgG or ACE2-Fc treatment for 24–48 h. Image documentation highlighted the impact of Spike variants on cell fusion.

### Determination of IL-6 and TNF-α levels

We evaluated the levels of IL-6 and TNF-α in culture media using an ELISA assay (R&D Systems), following exposure to supernatants from H1650-ACE2 cells that were either transfected with a spike plasmid or infected with SARS-CoV-2 for 48 h. Human PBMCs (1 × 10^5^ cells/well) were incubated in a 96-well plate with these supernatants for 24 h. Subsequently, we collected the media and performed the ELISA according to the manufacturer's instructions. The absorbance at 450 nm for each sample was measured using a VersaMax microplate reader (Molecular Devices), facilitating the quantification of cytokine levels.

### Flow cytometry

We utilized FITC Labeling Kit (ab102884, Abcam) to conjugate ACE2-Fc with green fluorescent dye. 293 T cells, transfected with 5 µg of pcDNA3.1-Spike variant plasmids using Lipofectamine 3000 (Thermo Fisher Scientific) for 48 h, were harvested using 0.48 mM EDTA. These cells were then incubated with FITC-conjugated ACE2-Fc or an isotype control (Thermo Fisher Scientific) on ice for 1 h. Following two washes and resuspension in cold PBS, the fluorescence intensity was measured using an Attune NxT flow cytometer (Thermo Fisher Scientific).

### Pseudotype SARS‐CoV‐2 virus neutralization assay

Following the production and purification techniques for the pseudotyped SARS-CoV-2 lentivirus outlined by Huang et al. (2020), we proceeded with a neutralization test. ACE2-Fc, initially at concentrations of either 20 µg/ml or 50 µg/ml, was diluted in a twofold series within the culture medium. This diluted ACE2-Fc was then incubated alongside the pseudovirus at 37 °C for one hour. Subsequently, this mixture was applied to HEK293T cells that express ACE2 and maintained under the same conditions for 48 h. The inhibition of viral entry by ACE2-Fc was quantitatively evaluated by measuring the luciferase activity in the cells, as per Promega's kit (E1501) instructions.

### Plasma clot formation assay

Fibrin polymerization was assessed by measuring turbidity in a plasma clot assay. Human citrated plasma (Sigma-Aldrich) was diluted 1:3 with 20 mM HEPES. Equal volumes of this plasma mixture and a solution containing 3 μM trimeric Spike protein in 20 mM HEPES with 137 mM NaCl were combined and incubated at room temperature for 15 min. Clotting was initiated by adding equal volumes of 0.25 U/ml thrombin (Sigma-Aldrich) and 40 mM CaCl2. For the ACE2-Fc treatment groups, trimeric Spike protein was pre-incubated for 15 min with ACE2-Fc alone, ACE2-Fc plus Spike antibody (Genetex), or ACE2-Fc plus the ACE2 catalytic inhibitor MLN-4760 (MedChemExpress), prior to mixing with plasma and initiating clot formation. The turbidity of the solution was measured at 340 nm every 15 s for 15 min using a VERSAMAX microplate reader (Molecular Devices).

### Plaque reduction assay

We prepared Vero E6 cells in 24-well plates using DMEM enriched with 10% FBS and antibiotics one day before the experiment. SARS-CoV-2 viruses, pre-treated with antibodies at 37 °C for one hour, were added to the cell layers for another hour. Following this incubation, we removed the virus-antibody mixtures and washed the cells with PBS. The cells were then covered with a 1% methylcellulose medium and incubated for 5 days. After incubation, cells were fixed with 10% formaldehyde overnight and stained with 0.5% crystal violet to visualize plaques. We calculated inhibition rates using the formula: [(1—(Virus Titer with ACE2-Fc/Virus Titer with hIgG)) × 100%]. The minimal concentration of antibodies required to reduce 50% of plaque numbers (EC50) was calculated by non-linear regression analysis of the dose–response curves generated from plaque reduction assays. The virus isolate used in the current study is hCoV-19/Taiwan/NTU13/2020 (GISAID: EPI_ISL_422415), hCoV-19/Taiwan/NTU92/2021 (GISAID:: EPI_ISL_3979387), hCoV-19/Taiwan/NTU142/2022 (GISAID:: EPI_ISL_13105945), hCoV-19/Taiwan/NTU280/2022 (GISAID:: EPI_ISL_15908767), hCoV-19/Taiwan/NTU286/2023 (GISAID:: EPI_ISL_18436063) and hCoV-19/Taiwan/NTU293/2023 (GISAID:: EPI_ISL_18278880). All of the experiments involving SARS-CoV-2 virus were performed in the Biosafety Level-3 Laboratory of the First Core Laboratory, National Taiwan University College of Medicine.

### Virus yield reduction assay

H1650-ACE2 cells were seeded in 24-well plates using DMEM supplemented with 10% FBS and antibiotics one day before the assay. The cells were then exposed to SARS-CoV-2 (MOI = 0.01), which had been pre-incubated with antibodies at 37 °C for one hour. Following viral exposure, the cells were incubated for an additional 24–48 h at 37 °C. After incubation, cytopathic effects (CPE) were recorded through imaging, and cell lysates were collected for viral protein expression analysis. The supernatant was harvested to determine the viral titer via plaque assay in Vero E6 cells and subsequently inactivated with 1% formaldehyde at 37 °C overnight. Viral inactivation was confirmed using a plaque assay before transferring the samples to a BSL-2 laboratory for cytokine analysis.

### Statistical analysis

Data are presented as the mean with standard deviation (SD). We used an unpaired, two-tailed Student’s t-test to compare continuous variables, adjusting for unequal variances as needed. If the experiment includes more than two groups, one-way ANOVA is used to calculate an overall p-value, followed by post-hoc Tukey’s test analysis for multiple comparisons. We considered results statistically significant if P < 0.05.

## Results

### The cell fusion capability induced by Spike protein is positively correlated with cytotoxicity and cytokine release

**Fig. 1:**
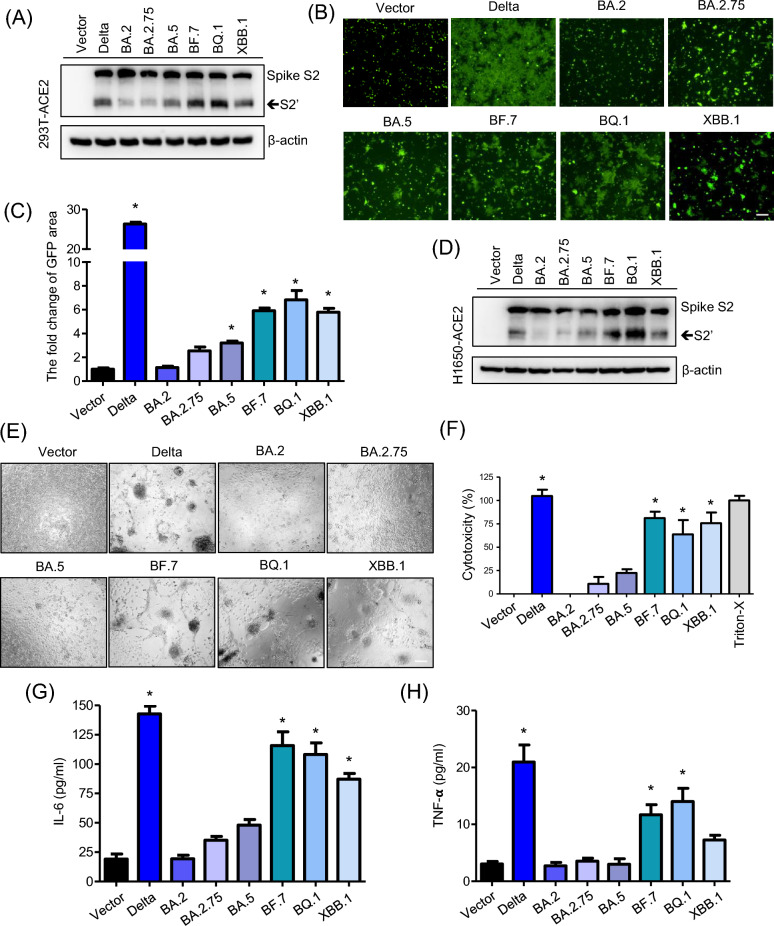
Varied properties of Spike protein variants. **A** The expression of different Spike variants in 293 T-ACE2 Cells. Two days after transfection, the presence of various Spike protein variants in cell lysates was confirmed by immunoblotting. β-actin was used as a loading control. **B** Cell Fusion in 293 T-ACE2 Cells. Images showing how different Spike protein variants induced cell fusion and syncytia formation. Scale bars represent 150 µm. **C** Quantitative syncytia analysis. The measured areas of syncytia formation to quantify the extent of cell fusion. **D** Levels of different Spike protein variants expressed in another cell line, H1650-ACE2. **E** Syncytia formation in H1650-ACE2 Cells. Similar to panel B, but demonstrating syncytia formation in H1650-ACE2 cells. Scale bars represent 150 µm. **F** Cytotoxicity post-Spike transfection. The cytotoxic effects observed 48 h after transfecting different Spike variants into H1650-ACE2 cells, with Triton-X100 treated cells serving as the 100% cytotoxicity control. **G**, **H** Impact on PBMCs. After treating PBMCs for 24 h with supernatants from H1650-ACE2 cells transfected with various Spike variants for 48 h, the levels of inflammation markers IL-6 and TNF-α were measured. Data are presented as mean ± SD from three replicates. Statistical analysis was performed by One-Way ANOVA with multiple comparisons *P < 0.05

To explore how various Spike protein variants of SARS-CoV-2 induced host cell fusion and lead to severe illness, we conducted a study using 293 T-ACE2 cells. We transfected these cells with pcDNA3.1(+) plasmids expressing the Spike proteins from Delta, Omicron BA.2, BA.2.75, BA.5, BF.7, BQ.1, and XBB.1 variants and assessed Spike proteolytic processing using a S2 specific antibody. Our findings revealed that compared to the Delta variant, the Omicron BA.2, BA.2.75, and BA.5 variants exhibited reduced S2’ proteolytic processing in 293 T-ACE2 cells. However, relative to Omicron BA.2, the BF.7, BQ.1, and XBB.1 variants showed increased S2’ processing (Fig. 1A). To confirm the cell fusion capabilities of various Spike protein mutants, we conducted a standard syncytia formation assay. Delta, Omicron BA.2, BA.2.75, BA.5, BF.7, BQ.1, and XBB.1 Spike proteins were individually co-transfected with a GFP plasmid into 293 T cells, which served as the effector cells (293 T-S). 293 T cells expressing ACE2 were used as the target cells. The area of green fluorescence, indicating syncytia formation, was measured to quantify the SARS-CoV-2 Spike-mediated cell fusion (N = 5). After 24 h of co-incubation, we observed that the Delta variant significantly induced syncytia formation, whereas the syncytia formation abilities of Omicron BA.2, BA.2.75, and BA.5 were reduced. Notably, the syncytia formation capabilities of the BF.7, BQ.1, and XBB.1 spike variants were significantly increased (Fig. 1B, C). To confirm these observations, we transfected H1650-ACE2 lung epithelial cells with the same Spike variants and again assessed S2 subunit expression and syncytia formation. The patterns were similar: BF.7, BQ.1, and XBB.1 not only increased S2’ expression but also enhanced cell fusion, similar to what was observed with the Delta variant (Fig. 1D, E). Using a cytotoxic LDH release assay, we evaluated the cytotoxic effects driven by these Spike proteins. Compared to the Delta variant, the BA.2, BA.2.75, and BA.5 variants showed reduced cytotoxicity. However, the cytotoxicity levels of BF.7, BQ.1, and XBB.1 were restored (Fig. 1F), indicating a resurgence in the harmful effects associated with these variants. Furthermore, we analyzed cytokine responses by exposing human PBMCs cells to supernatants from the transfected H1650-ACE2 cells. The Delta variant induced the highest levels of these cytokines, whereas the BA.2, BA.2.75, and BA.5 variants showed lower abilities to induce these cytokines compared to Delta. Remarkably, the cytokine induction by BF.7, BQ.1, and XBB.1 was substantially higher, aligning with their increased cytotoxic effects (Fig. 1G, H). Our findings indicate that spike-induced cell fusion and cytotoxicity are positively correlated with increased levels of IL-6 and TNF-α, exhibiting correlation coefficients (r) ranging from 0.66 to 0.84 (Fig. S1).

### ACE2-Fc can broadly bind various Spike variants and effectively inhibit their pseudovirus infections

**Fig. 2:**
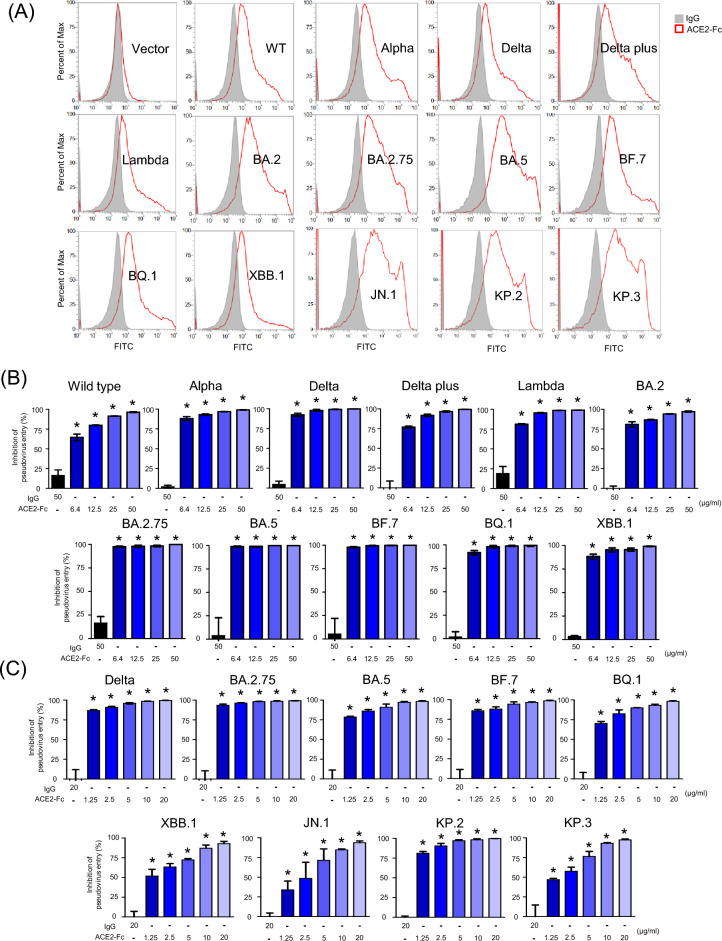
Blocking of pseudovirus entry by ACE2-Fc across different Spike variants. **A** Recognition by ACE2-Fc. Flow cytometry analysis showing the binding of ACE2-Fc, tagged with a fluorescent marker (FITC), to different Spike protein variants expressed on 293 T cells. Mouse IgG-FITC was used as an isotype control to validate the specificity of ACE2-Fc binding. **B**, **C** Inhibition of Pseudovirus Entry. These panels demonstrate the effectiveness of ACE2-Fc in blocking the entry of pseudoviruses into two types of cells: 293 T-ACE2 (**B**) and H1650-ACE2 (**C**). The results indicate that ACE2-Fc can prevent pseudovirus infection by interfering with the interaction between the Spike protein and the ACE2 receptor on the surface of target cells. Data are presented as mean ± SD from three replicates. Statistical analysis was performed by unpaired two-tail t-test. *P < 0.05

Our previous research highlighted the efficacy of the ACE2-Fc fusion protein, a humanized decoy receptor, in inhibiting viral entry and preventing infection by the wild-type (WT) strain of SARS-CoV-2 [16]. Motivated by these promising results, we aimed to evaluate whether ACE2-Fc could exert a similar inhibitory effect on various emerging SARS-CoV-2 variants. To this end, we initiated our study by engineering 293 T cells to express multiple Spike protein variants, employing flow cytometry to assess whether ACE2-Fc could effectively recognize and bind to these modified cells. Our experimental findings revealed a noticeable alteration in the binding pattern of ACE2-Fc compared to the standard IgG control. This was particularly evident in cells expressing not only the WT Spike protein but also those of several concerning variants, including Alpha, Delta, Delta plus, Lambda, BA.2, BA.2.75, BA.5, BF.7, BQ.1, and XBB.1. Additionally, ACE2-Fc has also shown the ability to recognize Spike proteins from the recently prevalent variants JN.1, KP.2, and KP.3. (Fig. 2A). These results suggest that ACE2-Fc retains broad efficacy against multiple variants by recognizing and binding to these altered Spike proteins. Further testing involved the creation of pseudotyped viruses by co-transfecting the cells with a lentiviral vector encoding luciferase alongside various Spike plasmids. These pseudoviruses were exposed to either ACE2-Fc or human IgG1 antibodies for one hour at 37 °C. Following this incubation, we introduced the pseudoviruses into 293 T-ACE2, observing the interactions for an additional 48 h. ACE2-Fc exhibited a dose-dependent inhibition of infection across a wide range of pseudoviruses, spanning from the WT to the XBB.1 variant, with effective concentrations ranging from 6.4 to 50 µg/ml (Fig. 2B). We also extended our investigation to lung cancer epithelial cells (H1650-ACE2) to explore the therapeutic potential of ACE2-Fc in a different cellular environment. Here, ACE2-Fc demonstrated significant protective effects at concentrations varying from 1.25 to 20 µg/ml, effectively preventing infection by pseudoviruses bearing Spike proteins from variants including Delta, BA.2, BA.2.75, BA.5, BF.7, BQ.1, XBB.1, JN.1, KP.2, and KP.3 (Fig. 2C). These findings highlight the versatile potential of ACE2-Fc to combat a wide range of SARS-CoV-2 variants.

### ACE2-Fc effectively inhibits cell fusion, cytotoxicity, and cytokine release induced by various Spike variants

**Fig. 3:**
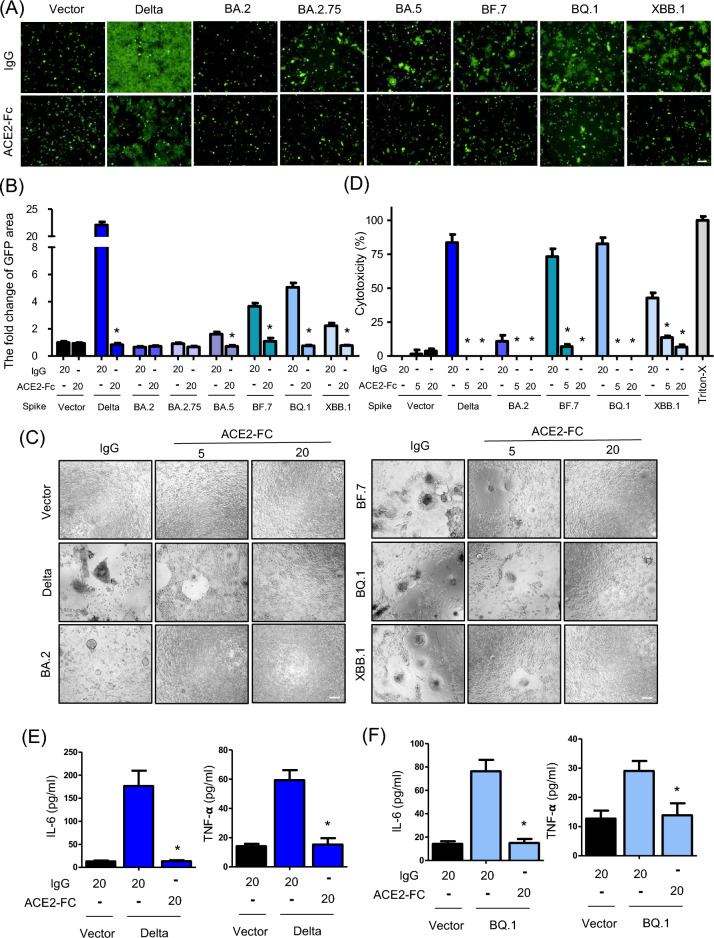
Inhibitory effects of ACE2-Fc on Spike-induced cell fusion and cytotoxicity. **A** Syncytia formation in 293 T-ACE2 Cells. ACE2-Fc inhibits the formation of syncytia induced by different Spike protein variants. The effectiveness of the inhibition is visually represented, with scale bars measuring 150 µm. **B** Quantitative analysis of GFP area. The quantitative results of the green fluorescent (GFP) area, which reflects the extent of syncytia formation. The calculations are based on a formula detailed in the Methods section of the study. **C** Syncytia reversal in H1650-ACE2 Cells. ACE2-Fc can reverse syncytia formation caused by different Spike variants in another cell type, H1650-ACE2. Scale bars represent 150 µm. **D** Reduction of cytotoxicity. ACE2-Fc reduces cytotoxicity observed 48 h after transfecting different Spike variants into H1650-ACE2 cells. **E**–**F** Reduction of cytokine induction. ACE2-Fc effectively reduces Delta and BQ.1 Spike meditated the induction of IL-6 and TNF-α in human PBMCs. Data are presented as mean ± SD from three replicates. Statistical analysis was performed by One-Way ANOVA with multiple comparisons (**B**, **D**) or unpaired two-tail t-test (**E**, **F**). *P < 0.05

The results from Fig. 1 show that cell fusion triggered by the spike protein is strongly linked to cell death and the induction of cytokines IL-6 and TNF-α. Consequently, we proceeded to investigate whether ACE2-Fc could inhibit cell fusion, cell death, and cytokine induction caused by different spike protein variants. In the syncytia formation assay, 293 T cells were transfected with 5 µg of various spike protein variants along with 0.5 µg of GFP for two days. These 293 T cells expressing spike proteins (293 T-S effector cells) were then treated with 20 µg/ml of either human IgG or ACE2-Fc for one hour. Following this treatment, the effector cells were co-cultured with 293 T cells expressing ACE2 (293 T-ACE2 target cells) for 24 h to observe the extent of green fluorescence, which indicates the level of cell fusion. The fusion events between effector and target cells were meticulously documented using a Zeiss Axiovert 200 M microscope, and the extent of syncytia formation was quantitatively analyzed using MetaMorph software. The results indicate that ACE2-Fc treatment significantly decreased syncytia formation in the Delta, BA.5, BF.7, BQ.1, and XBB.1 variants. In the control group and in groups where spike-induced cell fusion was not significant, such as with BA.2 and BA.2.75, ACE2-Fc treatment did not exhibit a noticeable effect (Fig. 3A, B). Next, we utilized lung epithelial cells H1650-ACE2 to confirm the effects of ACE2-Fc on spike-induced cell fusion, cytotoxicity, and cytokine induction. We transfected vectors for Delta, BA.2, BA.5, BF.7, BQ.1, and XBB.1 into H1650-ACE2 cells for 6 h, followed by treatment with 5 and 20 µg/ml ACE2-Fc and an incubation period of 48 h. The syncytia formation assay revealed that ACE2-Fc treatment can dose-dependently reverse spike-induced cell fusion. Notably, at 20 µg/ml, ACE2-Fc dramatically reversed syncytia formation in Delta, BF.7, BQ.1, and XBB.1. Even though BA.2 induced only trace amounts of cell fusion, ACE2-Fc was also effective in inhibiting this process (Fig. 3C). After the image acquisition, we collected supernatants from these experiments to perform cytotoxic LDH assay, aiming to quantify the reduction in spike-induced cytotoxicity. The results confirmed that ACE2-Fc significantly lowered cytotoxicity in a dose-dependent manner across all tested spike variants (Fig. 3D). Subsequently, we treated PBMCs with supernatants from cells expressing with the Delta and BQ.1 variants to observe the effects of ACE2-Fc on the induction of IL-6 and TNF-α. We found that ACE2-Fc significantly inhibited the induction of these cytokines mediated by the Delta and BQ.1 Spike proteins, compared to the control IgG group (Fig. 3E, F). This highlights ACE2-Fc's potential to mitigate inflammatory responses triggered by SARS-CoV-2 variants.

### ACE2-Fc can reduce cell entry, viral replication, and cytopathic effects for various SARS-CoV-2 strains

**Fig. 4:**
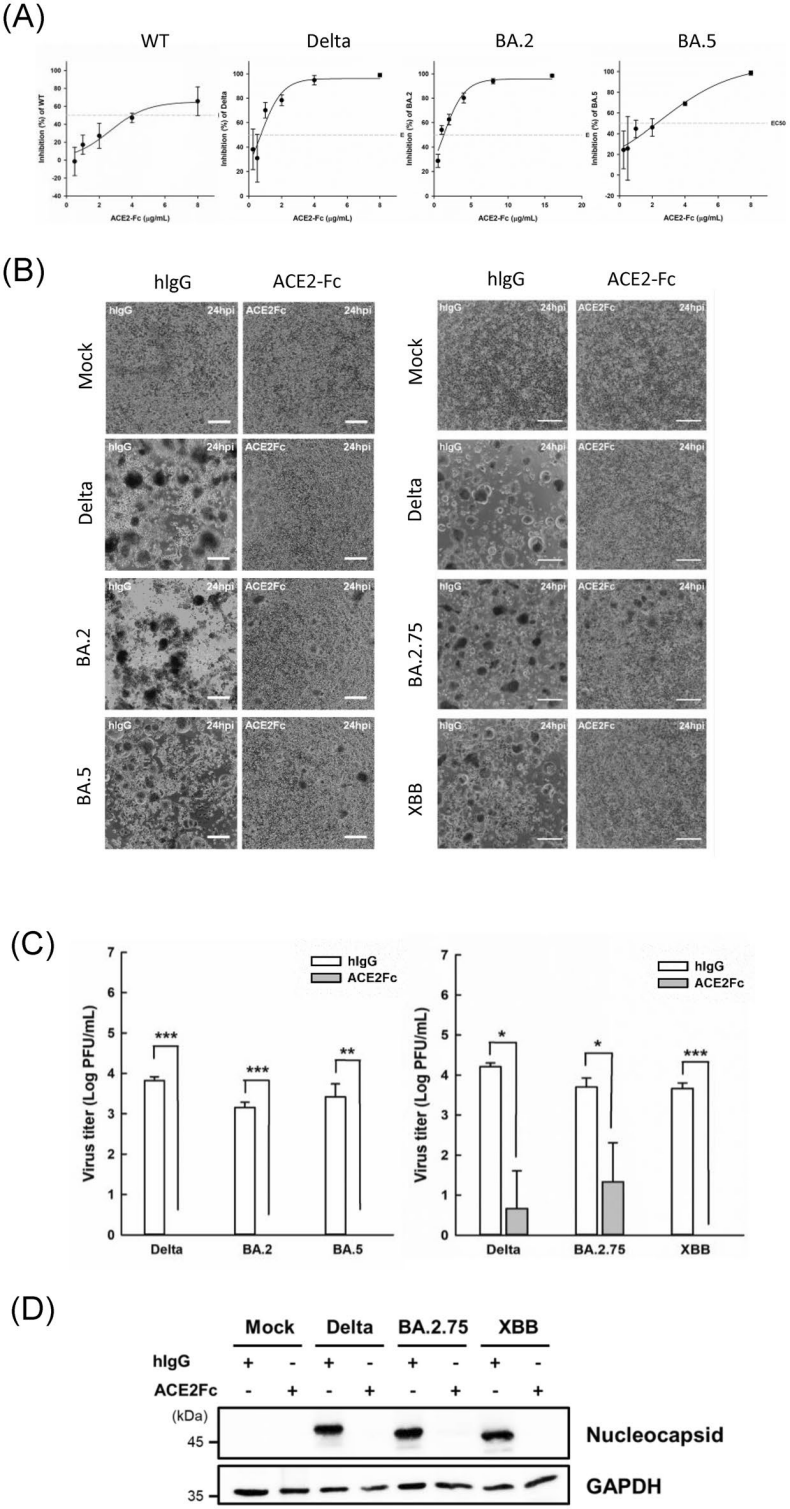
Inhibition of SARS-CoV-2 entry into host cells by ACE2-Fc. **A** Plaque assay inhibition. The ability of ACE2-Fc to inhibit infection by different SARS-CoV-2 variants using a plaque assay. The effectiveness of ACE2-Fc is compared to human IgG (hIgG), which serves as a baseline for inhibition. **B** Yield reduction assay. This assay was conducted to evaluate the inhibitory effects of ACE2-Fc on various coronavirus variants in H1650-ACE2 cells. The assay measures the reduction in the number of infectious virus particles as a result of ACE2-Fc treatment. **C** Plaque assay in Vero-E6 cells. This plaque assay quantifies the viral titer in the supernatant collected from the yield reduction assay. This method assesses the amount of virus that remains infectious after treatment with ACE2-Fc. Data are presented as mean ± SD from three replicates. Statistical analysis was conducted using an unpaired two-tail t-test. *P < 0.05, **P < 0.01, ***P < 0.001. **D** Inhibition of nucleocapsid protein expression. The effect of ACE2-Fc on the expression of the Nucleocapsid protein across different variants. The results show a reduction in Nucleocapsid protein levels, indicating effective inhibition of virus replication by ACE2-Fc

The effectiveness of the ACE2-Fc fusion protein against the coronavirus was confirmed through extensive testing with actual virus samples from COVID-19 patients treated at National Taiwan University Hospital. The pre-treatment of various SARS-CoV-2 strains with ACE2-Fc led to a significant, dose-dependent reduction in plaque formation in Vero E6 cells. This quantifiable decrease in viral replication is clearly documented in Fig. 4A. The effectiveness of ACE2-Fc varied notably among different viral strains, as evidenced by the half-maximal inhibitory concentrations (EC50). For the wild-type strain NTU13, the EC50 was recorded at 4.06 ± 0.39 μg/mL. The Delta variant NTU92 exhibited a lower EC50 of 1.76 ± 0.67 μg/mL, indicating a heightened sensitivity to ACE2-Fc treatment. The BA.2 variant NTU142 showed an even more pronounced sensitivity with an EC50 of 1.25 ± 0.23 μg/mL. In contrast, the BA.5 variant NTU280 required a slightly higher concentration, with an EC50 of 2.09 ± 0.67 μg/mL (Fig. 4A). Further validation of ACE2-Fc's inhibitory capability was demonstrated through yield reduction assays performed on H1650-ACE2 lung epithelial cells. Pre-treatment with ACE2-Fc markedly decreased the cytopathic effects (CPE) typically caused by five clinical SARS-CoV-2 strains: Delta (NTU92), BA.2 (NTU142), BA.5 (NTU280), BA.2.75 (NTU286), and XBB (NTU293) (Fig. 4B). The associated reduction in viral titers following treatment was quantified through a plaque assay conducted on Vero-E6 cells. The magnitude of viral reduction is compellingly shown in Fig. 4C, where a concentration of 20 μg/mL of ACE2-Fc was sufficient to significantly reduce the viral load for all evaluated strains. Moreover, the assay results detailed in Fig. 4D focused on the expression levels of the SARS-CoV-2 nucleoprotein, a critical component of the viral structure, in infected cells. The nucleoprotein levels for the Delta, BA.2.75, and XBB variants were substantially inhibited, reaffirming the potent antiviral action of ACE2-Fc across diverse viral variants.

### ACE2-Fc reduces cytotoxicity and cytokine release caused by CPE, and plasma clot formation induced by the Spike protein

**Fig. 5:**
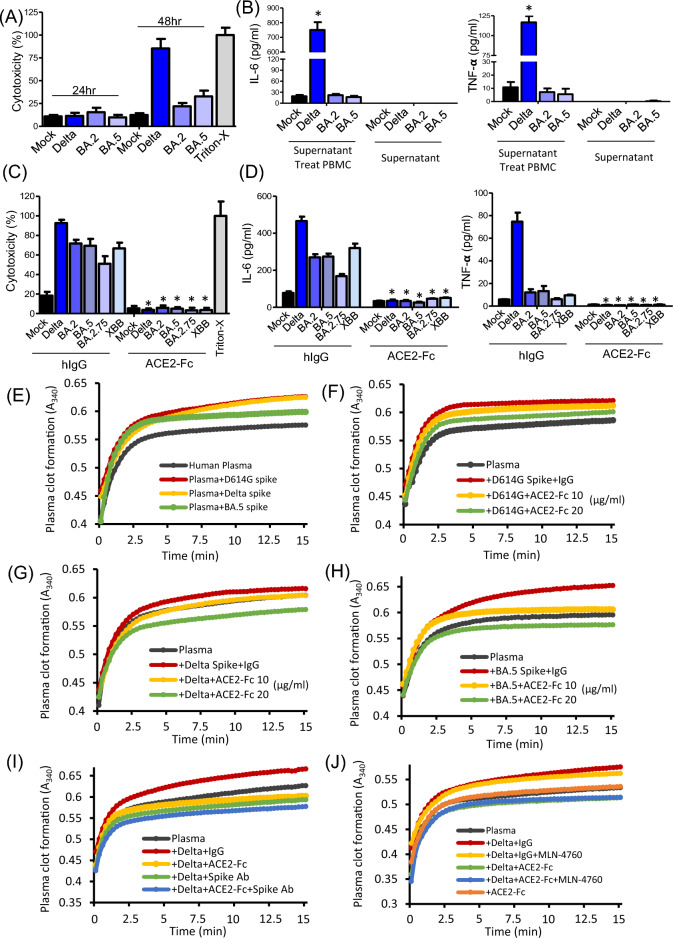
Blocking of SARS-CoV-2 induced cytotoxicity, cytokine release and clot formation by ACE2-Fc. **A** The cytotoxicity in H1650-ACE2 cells. The cytotoxic effects observed in cells infected with different SARS-CoV-2 variants over 24 and 48 h. After the infection period, the supernatant was collected to measure cell damage, using Triton-X100 treated cells serving as the 100% cytotoxicity control. **B** Cytokine levels in PBMCs post-infection. After 48 h of infection in H1650-ACE2 cells, the supernatants were used to treat PBMCs, and the levels of IL-6 and TNF-α were measured. *Significant differences compared to the MOCK group. P < 0.05. **C** Inhibition of cytotoxicity by ACE2-Fc. Pre-treatment with ACE2-Fc significantly reduces the cytotoxic effects in H1650-ACE2 cells infected with various coronavirus variants 48 h post-infection. **D** Reduction of cytokine release by ACE2-Fc. ACE2-Fc treatment effectively decreases the release of IL-6 and TNF-α by PBMCs that were exposed to supernatants from infected H1650-ACE2 cells. Data are presented as mean ± SD from three replicates. Statistical analysis was performed by One-Way ANOVA with multiple comparisons *P < 0.05. **E** The turbidity of human plasma clot formation with D614G, Delta, and BA.5 spike. **F**–**H** The effect of ACE2-Fc treatment on plasma clot formation induced by the D614G (**F**), Delta (**G**), and BA.5 **H** Spike proteins. **I** Effect of ACE2-Fc co-treatment with Spike antibody on plasma clot formation. (**J**) Effect of ACE2-Fc co-treatment with the ACE2 catalytic inhibitor MLN-4760 on plasma clot formation. **E**–**J** show representative results, with similar trends observed in three independent experiments

Next, we will further investigate the relationship between the CPE induced by SARS-CoV-2 and subsequent cytotoxicity and cytokine release. For this purpose, we will collect culture supernatants from H1650-ACE2 cells infected with various SARS-CoV-2 strains at either 24 or 48 h post-infection. These supernatants will be analyzed for LDH levels, providing an indication of the cytotoxicity associated with each viral strain. At 24 h post-infection, we observed negligible cytotoxicity across all virus strains. By 48 h, cytotoxicity had markedly increased in cells infected with the Delta strain. Conversely, cells infected with the BA.2 and BA.5 strains, which exhibited lower levels of CPE, showed only minor increases in cytotoxicity (Fig. 5A). We extended our analysis by collecting supernatants at 48hpi and then exposing human PBMCs to these supernatants for another 24 h. Our goal was to check for inflammation markers, IL-6 and TNF-α, in both the original cell culture supernatants and the supernatants after PBMCs exposure. Interestingly, although we initially detected no IL-6 or TNF-α, after exposing the PBMCs, we observed a significant increase in these markers in samples from cells infected with the Delta strain. However, infections with the BA.2 and BA.5 strains did not significantly change the levels of these cytokines (Fig. 5B). To explore the therapeutic potential of ACE2-Fc, we treated cells with this fusion protein before exposing them to the virus. The results were promising: ACE2-Fc significantly reduced cytotoxicity across all strains, including Delta, BA.2, BA.5, BA.2.75, and XBB, when compared to cells that received no such treatment (Fig. 5C). Furthermore, ACE2-Fc also effectively mitigated the increases in IL-6 and TNF-α induced by virus-related cell damage (Fig. 5D). In 2024, Ryu et al. demonstrated that the WT SARS-CoV-2 Spike protein binds with fibrin, leading to the formation of pro-inflammatory clots that contribute to systemic thromboinflammation and neuropathology in COVID-19 [26]. Building on this discovery, we aimed to assess whether Spike proteins from different variants also promote plasma clot formation and to evaluate the inhibitory potential of ACE2-Fc. We incubated recombinant trimeric spikes of D614G, Delta, and BA.5 variants with plasma from healthy donors and thrombin. We observed increased clot formation for all tested spikes, with the BA.5 spike inducing a smaller increase than D614G and Delta (Fig. 5E). Subsequently, we tested whether ACE2-Fc could reduce this clot formation. Our results indicated that ACE2-Fc at doses of 10 and 20 µg/ml effectively decreased clot formation in a dose-dependent manner, with significant inhibitory effects at 20 µg/ml, particularly against the Delta and BA.5 variants, even surpassing the control levels (Fig. 5F–H). To further clarify the mechanism by which ACE2-Fc inhibits clot formation, we co-treated human plasma with Delta Spike protein and either a Spike antibody or an ACE2 catalytic inhibitor combined with ACE2-Fc. As shown in Fig. 5I, treatment with 10 μg/ml Spike antibody alone was sufficient to inhibit clot formation. Co-treatment with 10 μg/ml ACE2-Fc and Spike antibody could induced an even stronger inhibitory effect. In contrast, co-treatment with the ACE2 catalytic inhibitor MLN-4760 and ACE2-Fc did not result in any significant difference (Fig. 5J). Additionally, no difference was observed between the ACE2-Fc–treated group without Spike and the plasma-only control group (Fig. 5J). These findings suggest that ACE2-Fc inhibits Spike-induced clot formation may primarily by blocking Spike binding, rather than through its enzymatic activity.

**Fig. 6:**
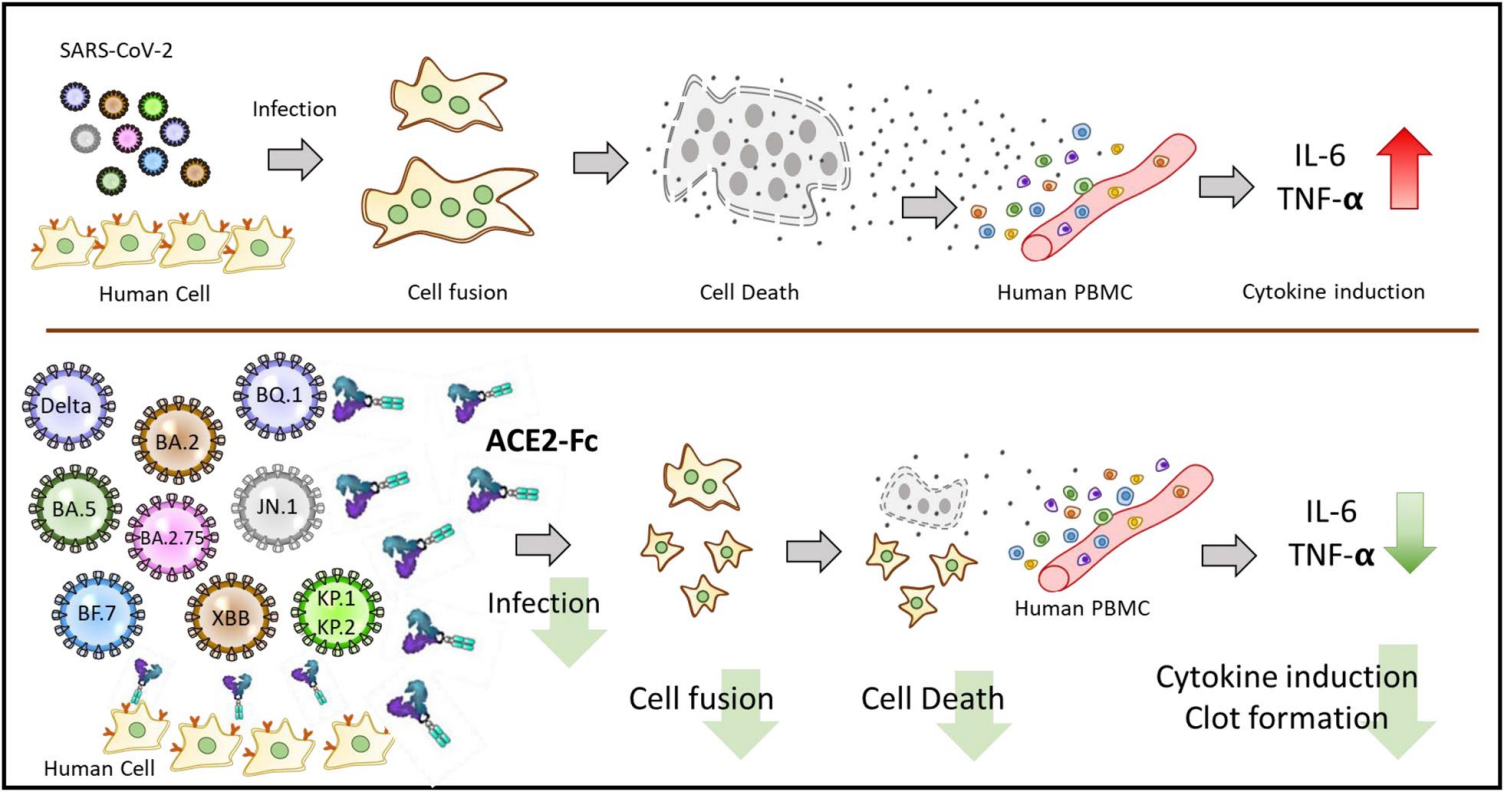
Schematic diagram of the impact of SARS-CoV-2 variants and ACE2-Fc inhibition. This diagram illustrates the process of cell fusion induced by different SARS-CoV-2 variants, which leads to cytotoxicity and cytokine induction in host cells. It also shows how ACE2-Fc treatment can inhibit these effects. ACE2-Fc is highlighted as a therapeutic agent that effectively blocks the disease progression at multiple stages: preventing the virus from entering cells, reducing cell fusion, mitigating cell damage, and decreasing the release of inflammatory cytokines

These findings underscore the dual protective effects of ACE2-Fc against both the direct cytotoxic consequences of SARS-CoV-2 infection and the associated inflammatory responses (Fig. 6), providing valuable insights into its potential as a therapeutic agent in managing COVID-19 by not only curbing viral entry but also attenuating the inflammatory sequelae of the infection. This study sets the stage for further investigations into the mechanistic pathways of SARS-CoV-2 induced cellular damage and thromboinflammation, potentially can guide more effective therapeutic strategies.

## Discussion

Since its emergence in late 2019, the COVID-19 pandemic remains a significant global health challenge [27]. According to the WHO update in February 2025, SARS-CoV-2 has resulted in over 777 million infections and 7 million deaths worldwide, ranking it among the deadliest viruses in human history [28]. The virus has evolved over time, leading to the emergence of new variants with differing impacts on public health [15, 29]. Initially, the original wild-type and Delta variants were associated with higher severity and mortality rates [30, 31]. However, the subsequent Omicron variant, while less severe, showed a marked increase in transmissibility [32–34]. The severity of COVID-19 varies among different variants, but the underlying mechanisms of these variations are not yet fully understood. This study showed that different variants of the Spike protein cause varying levels of cell fusion and formation of syncytia, which are linked to cytotoxicity and inflammation. We observed that the Delta variant of the Spike protein is more effective at causing cell fusion, leading to increased cytotoxicity, while the Omicron BA.2 variant results in less cell fusion and consequently less damage. Experiments with actual viruses confirmed that the Delta variant causes more severe effects in cells, consistent with higher rates of severe illness and death observed in clinical [33]. In contrast, the Omicron BA.2 and BA.5 variants are associated with milder symptoms. However, it’s important to note that certain sub-lineages of Omicron, such as BF.7, BQ.1, and XBB, despite not being as potent as Delta, still show enhanced cell fusion, cell damage, and increased cytokine induction. Our findings suggest that the severity of illness caused by different SARS-CoV-2 variants may be closely related to the fusion capacity of their Spike proteins, which may contribute to increased cytotoxicity and cytokine release.

Persistent high levels of early response proinflammatory cytokines, including IL-6, TNF-α, and IL-1β, can trigger a cytokine storm in COVID-19 patients. This severe condition heightens the risk of vascular hyperpermeability and multiorgan failure, potentially leading to death [35–37]. Berghe et al. (2006) demonstrated that when cell membranes break down during necrosis, the subsequent release of cytoplasmic factors activates antigen-presenting cells and other immune cells. This activation further boosts the production and release of additional cytokines like IL-6 [38]. This study revealed that overexpressing various Spike proteins in 1650-ACE2 cells leads to cell fusion, causing the release of LDH and increased cytotoxicity. When supernatants from these cells were used to culture PBMCs, cytokines such as IL-6 and TNF-α were released. This response may be triggered by substances from dying cells, including cellular debris, perforin, or free Spike proteins [6, 39]. Niu et al. demonstrated that the Spike protein indirectly causes TNF-α production in natural killer (NK) cells, which then prompts monocytes to release IL-6 [40]. Ryu et al. in 2024 discovered that the Spike protein can bind with fibrin to form pro-inflammatory clots, leading to thromboinflammation and neuropathology [26]. In summary, SARS-CoV-2 spike protein induced host cell–cell fusion is likely one of the mechanisms of cellular damage, which may lead to cell death and cytotoxicity. Substances released from ruptured cells can further trigger the release of cytokines from immune cells, exacerbating the thromboinflammatory respons. Other mechanisms by which the Spike protein causes damage include activation of Toll-like receptor signaling, initiation of the NF-κB pathway, stimulation of the NLRP3 inflammasome, promotion of M1 macrophage polarization leading to apoptosis, and induction of autophagy through the PI3K/AKT/mTOR pathway [41–45]. These processes can lead to elevated production of proinflammatory cytokines and the systemic release of inflammatory mediators, potentially resulting in a cytokine storm. Several studies have demonstrated that the structural proteins of SARS-CoV-2, including Spike, Envelope, Membrane, and Nucleocapsid, can each independently induce cellular damage [46]. Our findings also indicate that the Spike protein alone may possess pathogenic properties independent of the intact virus. These observations raise concerns about the use of the Spike as an immunogen in vaccine development and suggest that such strategies should be approached with caution.

Despite advances in therapeutic antibodies, the continuous mutation of the SARS-CoV2 virus poses a significant challenge, as these antibodies often fail to neutralize new variants. The rapid mutation rate has made many previously developed drugs ineffective [14, 47, 48]. While most current SARS-CoV-2 strains cause only mild, flu-like symptoms, the potential emergence of highly virulent variants remains a concern [49]. Therefore, developing a broad-spectrum drug is crucial, serving both as a treatment and a preventative measure against possible future SARS outbreaks. This study highlights the effectiveness of the using natural receptor as decoy, ACE2-Fc, which can broadly recognize a wide range of mutant Spike proteins, including wild type, Alpha, Delta, Delta plus, Lambda, BA.2, BA.2.75, BA.5, BF.7, BQ.1, XBB.1, JN.1, KP.2, and KP.3. ACE2-Fc effectively reduces pseudovirus infections caused by all of these Spike variants, underscoring its potential as a wide-ranging therapeutic agent. Most importantly, using host nature receptor as decoy, the ACE2-Fc can inhibit cell fusion initiated by various Spike proteins, effectively reducing subsequent cytotoxicity and the release of IL-6 and TNF-α from PBMCs. Additionally, ACE2-Fc has been demonstrated to reduce the D614G, Delta, and BA.5 spike-induced plasma clot formation. This suggests that ACE2-Fc could also mitigate SARS-CoV-2 infection-caused thrombosis. In experiments with the actual virus, ACE2-Fc effectively reduced the CPE induced by Delta, BA.2, BA.5, BA.2.75, and XBB. It also lowered the viral titer of newly formed viruses in infected cells and decreased nucleoprotein expression. ACE2-Fc additionally reduces cytotoxicity following infection with different virus strains and inhibits the post-infection release of IL-6 and TNF-α from PBMCs. These results suggest that ACE2-Fc is effective against rapidly mutating SARS-CoV-2 strains and offers a promising solution to combat viral immune evasion.

## Conclusions

The SARS-CoV-2 outbreak led to an unprecedented global pandemic and widespread lockdowns across nations. Although the situation with SARS-CoV-2 has now stabilized, ongoing research is crucial to prevent future outbreaks. This study demonstrates that the Spike protein itself possesses pathogenic properties, causing cytotoxicity and promoting cytokine release. ACE2-Fc can bind to various Spike proteins to inhibit infection, cell fusion, cytotoxicity, cytokine release, and clot formation, demonstrating its effectiveness against rapidly mutating viruses. As long as SARS-CoV-2 enters the human body through ACE2 receptor, ACE2-Fc could serve as a potent solution to combat immune evasion.

## Supplementary Information

Supplementary Material 1.

## Data Availability

Data and materials are available from the corresponding authors upon reasonable request.

## Abbreviations

r: Correlation coefficients
CPE: Cytopathic effects
LDH: Lactate dehydrogenase
NTU: National Taiwan University
PBMCs: Peripheral blood mononuclear cells
RBD: Receptor binding domain
S: Spike
EC50: The half-maximal inhibitory concentrations
WT: Wild-type
