# Supplementary material for: The ACE2 decoy receptor can overcome immune escape by rapid mutating SARS-CoV-2 variants and reduce cytokine induction and clot formation
Source: J Biomed Sci. 2025 Jun 26;32:59. doi: 10.1186/s12929-025-01156-4 (PMC12199494; doi:10.1186/s12929-025-01156-4)

**Fig. S1** The correlation between cell fusion, cytotoxicity, and cytokine release in Fig. 1. (A) The correlation between cell fusion and cytotoxicity. (B) The correlation between cell fusion and IL-6 release. (C) The correlation between cell fusion and TNF-α release. (D) The correlation between cytotoxicity and IL-6 release. (E) The correlation between cytotoxicity and TNF-α release. r: correlation coefficients


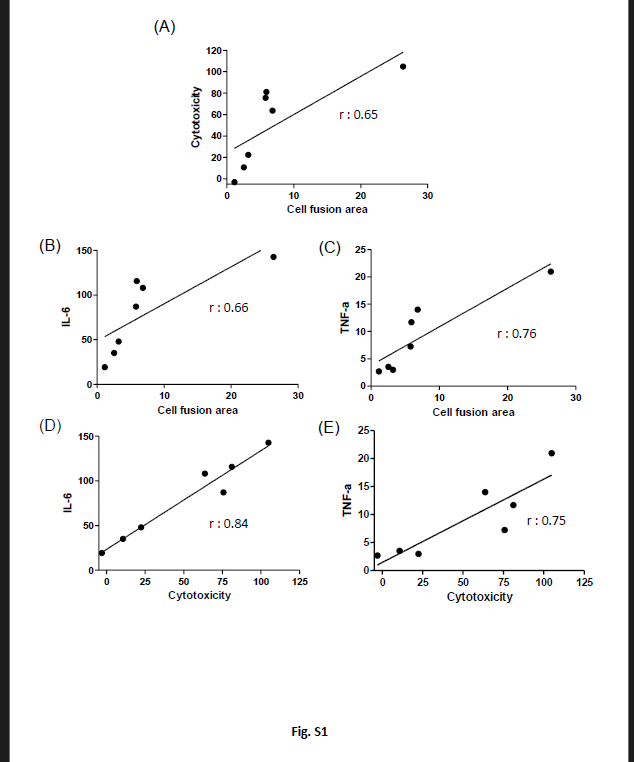

Supplement: Supplementary file 1 — Supplementary Material 1. [file 12929_2025_1156_MOESM1_ESM.docx]
